# Nanobody-armed T cells endow CAR-T cells with cytotoxicity against lymphoma cells

**DOI:** 10.1186/s12935-021-02151-z

**Published:** 2021-08-24

**Authors:** Hongxia Wang, Liyan Wang, Yanning Li, Guangqi Li, Xiaochun Zhang, Dan Jiang, Yanting Zhang, Liyuan Liu, Yuankui Chu, Guangxian Xu

**Affiliations:** 1 General Hospital of Ningxia Medical University, School of Clinical Medicine, Ningxia Medical University, Yinchuan, China; 2 Guangdong Provincial Key Laboratory of Medical Molecular Diagnostics, School of Medical Technology, Institute of Clinical Laboratory, Guangdong Medical University, Dongguan, China; 3 General Hospital of Ningxia Medical University, Yinchuan, China

**Keywords:** CAR-T, mAb, Nanobody, CD19, CD20

## Abstract

**Background:**

Taking advantage of nanobodies (Nbs) in immunotherapy, we investigated the cytotoxicity of Nb-based chimeric antigen receptor T cells (Nb CAR-T) against lymphoma cells.

**Methods:**

CD19 Nb CAR-T, CD20 Nb CAR-T, and Bispecific Nb CAR-T cells were generated by panning anti-human CD19- and CD20-specific nanobody sequences from a natural Nb-expressing phage display library, integrating Nb genes with a lentiviral cassette that included other CAR elements, and finally transducing T cells that were expanded under an optimization system with the above generated CAR lentivirus. Prepared Nb CAR-T cells were cocultured with tumour cell lines or primary tumour cells for 24 h or 5 days to evaluate their biological function.

**Results:**

The nanobodies that we selected from the natural Nb-expressing phage display library had a high affinity and specificity for CD19 and CD20. CD19 Nb CAR-T, CD20 Nb CAR-T and Bispecific Nb CAR-T cells were successfully constructed, and these Nb CAR-T cells could strongly recognize Burkitt lymphoma cell lines (Raji and Daudi), thereby leading to activation, enhanced proliferation, and specific killing of target cells. Furthermore, similar results were obtained when using patient samples as target cells, with a cytotoxicity of approximately 60%.

**Conclusions:**

Nanobody-based CAR-T cells can kill both tumour cell lines and patient-derived tumour cells in vitro, and Nb-based CAR-T cells may be a promising therapeutic strategy in future immunotherapy.

**Supplementary Information:**

The online version contains supplementary material available at 10.1186/s12935-021-02151-z.

## Background

Cancer immunotherapy has shown excellent clinical therapeutic effects against many cancers, CAR-T cell therapy is one of the most promising immunotherapy approaches [1–6]. The FDA has already approved five drugs (Kymriah, Yescarta, Tecartus and Breyanzi targeting CD19 and Abecma targeting BCMA) to treat B-cell precursor acute lymphoblastic leukaemia (B-ALL), R/R large B-cell lymphoma, R/R mantle-cell lymphoma or multiple myeloma [7–11]. Classical CARs consist of three parts: an extracellular antigen recognition region composed of single chain variable fragment (scFv), a transmembrane domain like CD8a, intracellular activation domains including costimulatory molecules 4-1BB and/or CD28 and a CD3ζ signaling domain [12–14]. The most widely studied and mature CAR is 2nd-generation CAR. ScFv is usually derived from a monoclonal antibody (mAb), which consists of a heavy-chain variable fragment connected to a light-chain variable fragment by a flexible linker [15, 16]. Guedan et al. reported that scFv often leads to recurrence in some patients, which is due to its large size, high immunogenicity, weak affinity, easy aggregation, tonic signaling, and often not fold efficiency [17–20]. Therefore, it is imperative to introduce alternative antibodies that enhance the efficiency of CAR-T cell treatment.

Recent studies have shown that nanobody-based CAR-T cells exert obvious antitumour effects [21–23]. nanobodies, also known as the variable domain of the heavy chain of heavy chain antibody (VHH), were first found in dromedaries by Hamers Castermans in 1993 and then identified in Camelidae and sharks. nanobodies belong to the variable region of the heavy chain antibodies (HcAbs), which only contain variable regions of heavy chain and CH2, and CH3 but are devoid of light chain and CH1 [24, 25]. Compared with mAbs that need six complementarity-determining regions (CDRs) to bind antigens, nanobodies only need three CDRs, and the affinity and specificity are similar [26]. In addition, most sequences with identity to the human VH gene family III result in weak immunogenicity, therefore, applying Nb as part of the antibody recognition of CAR-T cells may be safer than mAbs derived from mice [27]. More importantly, with a mature surface display platform, it is feasible to obtain several Nbs that recognize various epitopes of the same antigen, which is hindered by mAb [28]. Furthermore, Nbs have been applied in antibody-drug conjugates owing to their small molecular weight (15 kDa), stability and strong penetrating power [29–31]. Above all, nanobodies show promising therapeutic applications due to their favourable characteristics [32–36].

Additionally, several studies have reported that one possible reason for the poor prognosis of CAR-T therapy is antigen escape [37–39]. Based on the promise of this theory, some groups have shown that tandem CAR-T, bispecific CAR-T, or a mixture of two single targeted CAR-T cells, can decrease this phenomenon to some extent [40–43].

Here, we acquired Nbs that specifically bind human CD19 and CD20, optimized T cell activation and expansion conditions, and generated CD19 Nb CAR-T, CD20 Nb CAR-T and Bispecific Nb CAR-T cells that with the ability to accurately recognize tumour cell, followed by activation and proliferation, and these Nb CAR-T cells could also effectively protect against tumour cells in vitro. Furthermore, cytotoxicity was verified by primary patient-derived tumour cells of acute lymphoblastic leukaemia (PD-ALL).

## Methods

### Generation of nanobodies (Nbs)

Anti-human CD19 and CD20 nanobodies were selected from a Bactrian camel naïve phage display library. First, 500 µl of phage and 500 µl of prepared biotinylated CD19 or CD20 antigen (Shanghai Anyan, China) were added to a 1.5 ml centrifuge tube and rotated for 1 h at room temperature. Then, 50 µl of streptavidin magnetic beads (Invitrogen) was added and incubated for 30 min. The phage was eluted with 800 µl Tris-HCl, pH 2.7, after several times washes. Finally, the neutralized eluent was added to TG1 bacteria, incubated at 37 °C for 30 min, and plated on 2YT plates supplemented with 2% glucose, 100 µg/ml ampicillin, 50 µg/ml kanamycin and cultured overnight at 37 ℃. The colonies were collected, named in the first round of panning library, and stored in glycerine for later use. Except for the colonies that were retained by the precipitated phage, the second round of panning was carried out with the same methods as the first, followed by 4 repeats. Several single colonies from rounds 3 and 4 of panning were selected and identified. The identified Nb sequences were linked with IgG Fc, His-tags and then cloned into the pCZN1 vector. The purified Nbs were analysed by sodium dodecyl sulfate polyacrylamide gel electrophoresis (SDS–PAGE).

### Fluorescent semiconductor quantum dot (QD) immunofluorescence

Daudi cells were mounted on the cell slide by centrifugation at 1000 rpm for 15 min and fixed with 4% paraformaldehyde for 10 min. Next, the cells were washed with TBS, permeabilized with 0.1% Triton X-100 for 20 min, and blocked with 5% bovine serum albumin for 30 min. Then, the cells were stained with a 1:250 dilution of Nbs in TBS or TBS alone at 4 ℃ for 12 h, and next day, they incubated with a 1:200 dilution of biotinylated anti-His-tag secondary antibody at RT for 1 h, blocked again and incubated with QD-streptavidin for 30 min, followed by imaging under a fluorescence microscopy.

### Surface plasmon resonance (SPR)

For determination of the affinity of the obtained nanoantibodies, SPR was used. The experiment was performed using a Biacore T200 system (GE Healthcare Life Sciences). Mouse anti-human IgG (FC) antibodies were immobilized on a sensor chip (GE Healthcare) to capture the nanobody. Next, the nanobody was injected into the experimental channel at a flow rate of 10 µl/min. Finally, human CD19/CD20 antigen in 7 groups of serial twofold dilutions of antigen at 10 µg/ml was successively injected into the test channel and reference channel at a flow rate of 30 µl/min, an association time of 120 s, and a separation time of 300 s. The KD values were calculated using Biacore T200 software.

### Cell lines and patient samples

The following cell lines were used: K562 (a chronic myelogenous leukaemia line), Daudi, Raji (a Burkitt lymphoma cell line), and 293T (an embryonic kidney cell line). All cell lines were purchased from ATCC and maintained in RPMI 1640 supplemented with 10% foetal bovine serum (BI) and 1% penicillin-streptomycin (Solarbio), while 293T cells were cultured in DMEM. For generation of luciferase-expressing cell lines, wild-type tumour lines were transduced with a lentiviral vector encoding firefly luciferase, followed by puromycin selection of luciferase-positive cells for up to 14 days.

Human blood samples were acquired from the General Hospital of Ningxia Medical University and approved by the Ethics Committee of General Hospital of Ningxia Medical University. Primary ALL cells were obtained from diagnosed ALL patients, the expression of CD19 and CD20 was detected and the cells were cultured in RPMI 1640 with 10% FBS.

### CAR construction

For CD19 Nb CAR and CD20 Nb CAR, nanobodies were incorporated into basic CARs that were composed of IgG4 hinges, CD8 transmembrane domains, and 4-1BB and CD3ζ signaling domains. For Bispecific Nb CAR, the anti-CD20 and anti-CD19 nanobodies were linked by (EAAAK)_3_ and then combined with basic CARs. All CARs included a fluorescent protein tracer linked to a P2A sequence. Then, complete CAR sequences were cloned into a lentiviral plasmid backbone under the regulation of a human EF-1α promoter. The lentiviruses were produced by transfecting 293T cells and concentrated by ultracentrifugation. The concentrated CAR lentivirus was immediately stored at − 80 ℃.

### Western blot analysis

Proteins of 293T cells were harvested after 72 h of transduction, and then, western blots were performed using mouse anti-human CD3ζ primary antibody at 1:500 (Thermo).

### Primary T cell isolation, expansion and transduction

Peripheral blood derived from healthy donors were collected using heparin or ethylenediamine tetraacetic acid (EDTA) anticoagulant blood vessels was purified by density gradient centrifugation (TBD), and CD3-positive T cells were enriched by positive selection using magnetic bead separation (Miltenyi Biotec).

For optimization of T cell expansion conditions, isolated T cells were cultured in X-VIVO15 medium (Lonza) supplemented with 200 or 400 U/ml IL-2, with or without 10% FBS or autologous plasma, at a density of 1 × 10^6^ cells/ml and activated with immobilized 2 µg/ml anti-human CD3/CD28 antibody (Pepro Tech) or together with additional 5 µg/ml RetroNectin for 24 h, 48 h, and 72 h.

For transduction, a 48-well cell culture plate coated with 20 µg/ml RetroNectin (TaKaRa Bio) was used. First, according to multiplicity of infection (MOI) of 3 and the calculated relevant volume of virus, viruses were added to prepared 48-well plates and centrifuged at 32 °C (2000*g*, 2 h). Then, T cells were collected from the plates after 48 h of activation and centrifuged with viral supernatants at 32 ℃ (1000*g*, 1 h) in the presence of 8 µg/ml polybrene (Solarbio), 200 U/ml IL-2, 1% penicillin-streptomycin, followed by incubation overnight at 37 ℃, 5% CO2. After 24 h, an equal volume of medium was added, T cells were expanded at a density of 0.7–1 × 10^6^ cells/ml, and cell numbers were counted every 2 days. CAR expression was detected by flow cytometry, and expanded cells were used for the in vitro assay on day 10.

### Flow cytometry

For tumour cells expressing CD19 and CD20, 5 × 10^5^ tumour cells were harvested and washed twice with PBS. Then, the tumour cells were stained with 20 µl of APC-conjugated mouse anti-human CD19 (BD) and 20 µl of PE-conjugated mouse anti-human CD20 (BD) at room temperature and protected from light for 30 min, washed with PBS once, and resuspended in FACS buffer for assessment. For CAR expression, T cells were stained with PE-conjugated mouse anti-human CD3, and CAR-T cells were gated on FITC + PE+. For analysis of viability and the CD8/CD4 ratio, T cells were stained with 7-AAD, APC-Cy7TM-conjugated mouse anti-human CD8, and BV510-conjugated mouse anti-human CD4 (BD). For T cell activation, after overnight incubation at an E:T of 2:1, CD69 was detected using a PE-conjugated CD69 antibody (Biolegend). All data were recorded using a FACSCelesta™ Flow cytometer and analysed by FlowJo 10 software.

### CAR-T cell proliferation

For proliferation assays, tumour cells were treated with 20 µg/ml mitomycin C (MCE) for 12 h. CAR-T cells were labelled with 1 µM CellTrace Far Red (Life Technologies) in PBS at 37 °C for 20 min, 4–5-fold complete medium was added, and some CAR-T cells were collected for flow cytometry analysis after 1 h of incubation. Then, labeled T cells were cocultured with treated target cells at a 2:1 effector/target ratio in X-VIVO 15 medium without IL-2 for 5 days, followed by flow cytometric analysis, CAR T cells were gated on GFP signals.

### ELISA

CAR-T cells and tumour cells were cocultured at a ratio of 2:1 in X-VIVO medium without additional cytokines in 24-well plates. After 24 h, the supernatant was collected and used for IL-2 ELISA measurements (Biolegend).

### Cytotoxicity assay

For tumour cell lines, cytotoxicity was evaluated by luciferase assays (Promega). CAR-T cells and tumor cells were cocultured at a ratio of 2:1 for 24 h in 96-well plates.The same volume of assay buffer was added tothe wells and incubated for 10 min at room temperature, and then, luminescence values were obtained by a microporous plate detector. The percentage of specific lysis was calculated by the following formula: $1-\left(\frac{\text{expriment values}}{\text{max values}}\right)\times 100\%.$

For tumour cells derived from ALL patients, cytotoxicity was evaluated by lactate dehydrogenase (LDH,Dojindo). Primary ALL cells were isolated through gradient centrifugationand then some cells were used to test the expression of CD19 and CD20 by flow cytometry. Primary ALL cells were incubated with CAR-T cells for 18 h, and the experimental stetting was based on the instructions of the test kits. Before addition of assay buffer, lysis buffer was added intarget maximum release and volume correction control wells. After 30 min incubation at roomtemperature, the OD490 nm was obtained by a Thermo Scientific Microplate Reader. The percentage of specific lysis was calculated by the following formula: $\left(\frac{\text{Experimental - Effector Spontaneous - Target Spontaneous}}{\text{Target Maximum Release - Target Spontaneous}}\right)\times 100\%.$

### Statistical analysis

All statistical analyses were performed using SPSS 26.0 and GraphPad Prism version 7. Data variance was analysed by one-way ANOVA. *p < 0.05, **p < 0.01, ***p < 0.001, ns, not significant (P > 0.05). All data are shown as the mean ± SD of three independent replicates.

## Results

### Select Nbs that have a high affinity for human CD19 and CD20

**Fig. 1:**
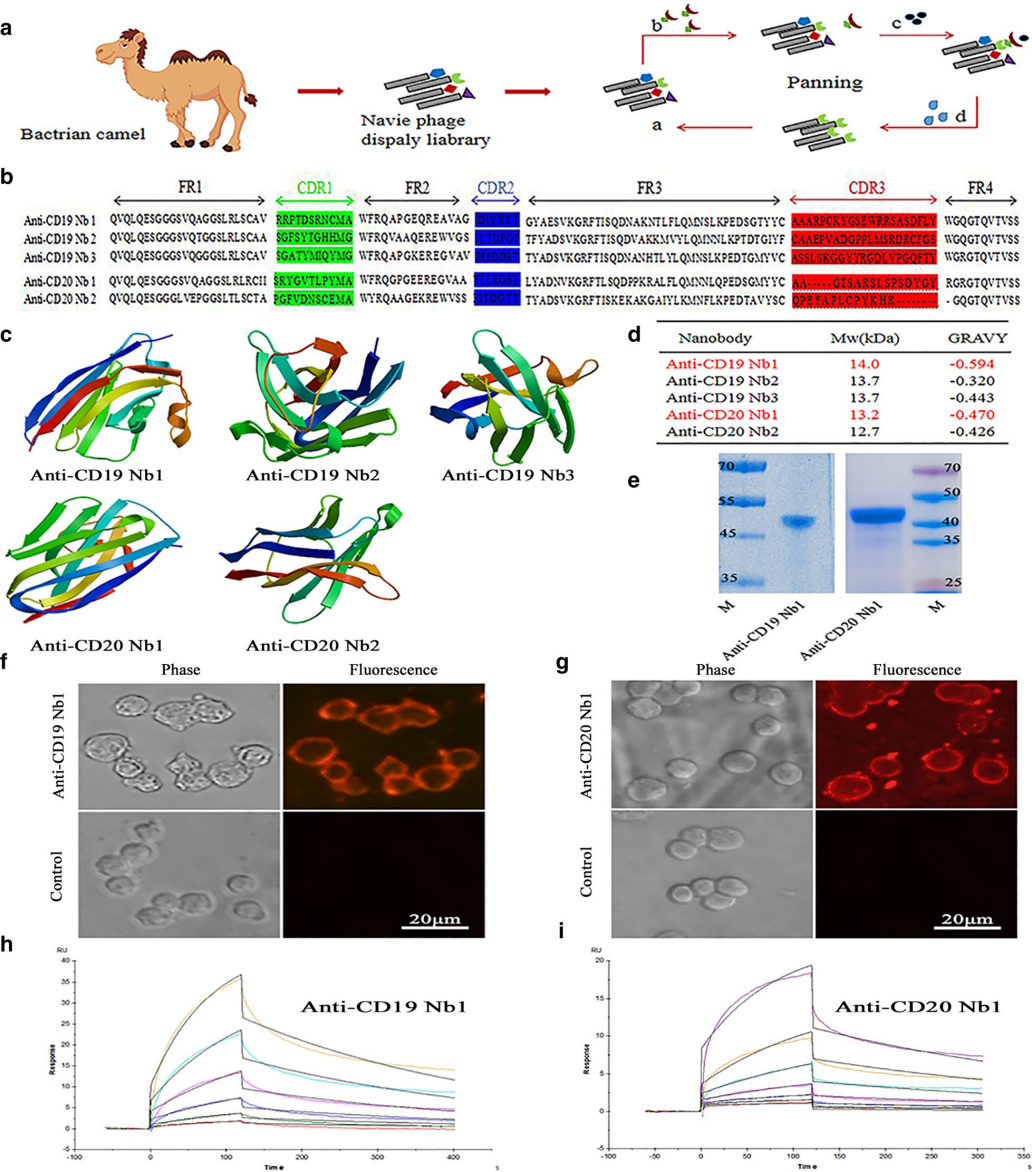
Generation of Nbs. **A **Schematic diagram of the generation of the Nbs. First, a nanobody library was prepared to obtain Nbs (*a*), and then, human CD19 or CD20 was added to bind the Nb (*b*). Finally, streptomycin-labeled magnetic beads were mixed with those complexes (*c*), followed by several washes (**d**). A total of 4 rounds of panning was performed. **B** Amino acid sequence of anti-CD19 and anti-CD20 Nbs. The framework (FR) and complementarity-determining region (CDR) sequences were defined according to the IMGT. CDR1, CDR2 and CDR3 are highlighted in green, blue and red, respectively. **C** Simulation of the three-dimensional crystal structure of anti-CD19 Nb1-3 and anti-CD20 Nb1-2 using Swiss-model. **D** Nb’s molecular weight (Mw) and grand average of hydropathicity (GRAVY) were analysed by the online soft ExPAsy-ProtParam Tool. **E** SDS–PAGE analysis of the molecular weights of the purified anti-CD19 Nb1 and anti-CD20 Nb1 antibodies. **F**, **G** Cell binding capacity of nanobodies detected by QD immunofluorescence. Fluorescence was captured by fluorescence microscope, and TBS was used as a control. **H**, **I** The affinity between CD19/CD20 and Nbs was determined by SPR binding assays

To obtain nanobodies that specifically target human CD19 and CD20, we adopted magnetic bead-based selection (Fig. 1A). After 4 rounds of panning, we identified three nanobodies that bound CD19 and two nanobodies that bound CD20. The sequences in the CDR3 domain were absolutely different, indicating that these Nbs could bind distinct regions of CD19 and CD20 (Fig. 1B). The 3D structure was also simulated (Fig. 1C). To determine the best sequence for CAR construction, we first assessed the molecular weight and grand average of the hydropathicity of each sequence because hydrogen bond interactions influence the affinity between antigen and antibody. Preliminary analysis indicated that anti-CD19 Nb1 and anti-CD20 Nb1 have the highest values; therefore, we utilized these two sequences in all subsequent experiments (Fig. 1D). Then, Nbs were expressed and purified, and an obvious band of approximately 50 kDa was detected by SDS–PAGE, which is consistent with the expected molecular size (Fig. 1E). Furthermore, taking advantage of QD immunofluorescence in imaging, we used QDs to analyse the cell binding capacity of Nbs. We found that only in the presence of Nbs was red fluorescence observed on the surface of the Daudi cells, while with no specific fluorescence was observed when TBS was used as a control (Fig. 1F, G). Finally, we tested the affinity of these nanobodies by SPR. SPR revealed that the dissociation rate constant (KD) of CD19 was 0.29 µM and that of CD20 was 0.147 µM, indicating the high binding ability to CD19 and CD20 (Fig. 1H, I). Taken together, these results indicate that we selected specific nanobody sequences for the generation of CARs.

### Generation of CD19, CD20, and Bispecific Nb CAR-T cells

**Fig. 2:**
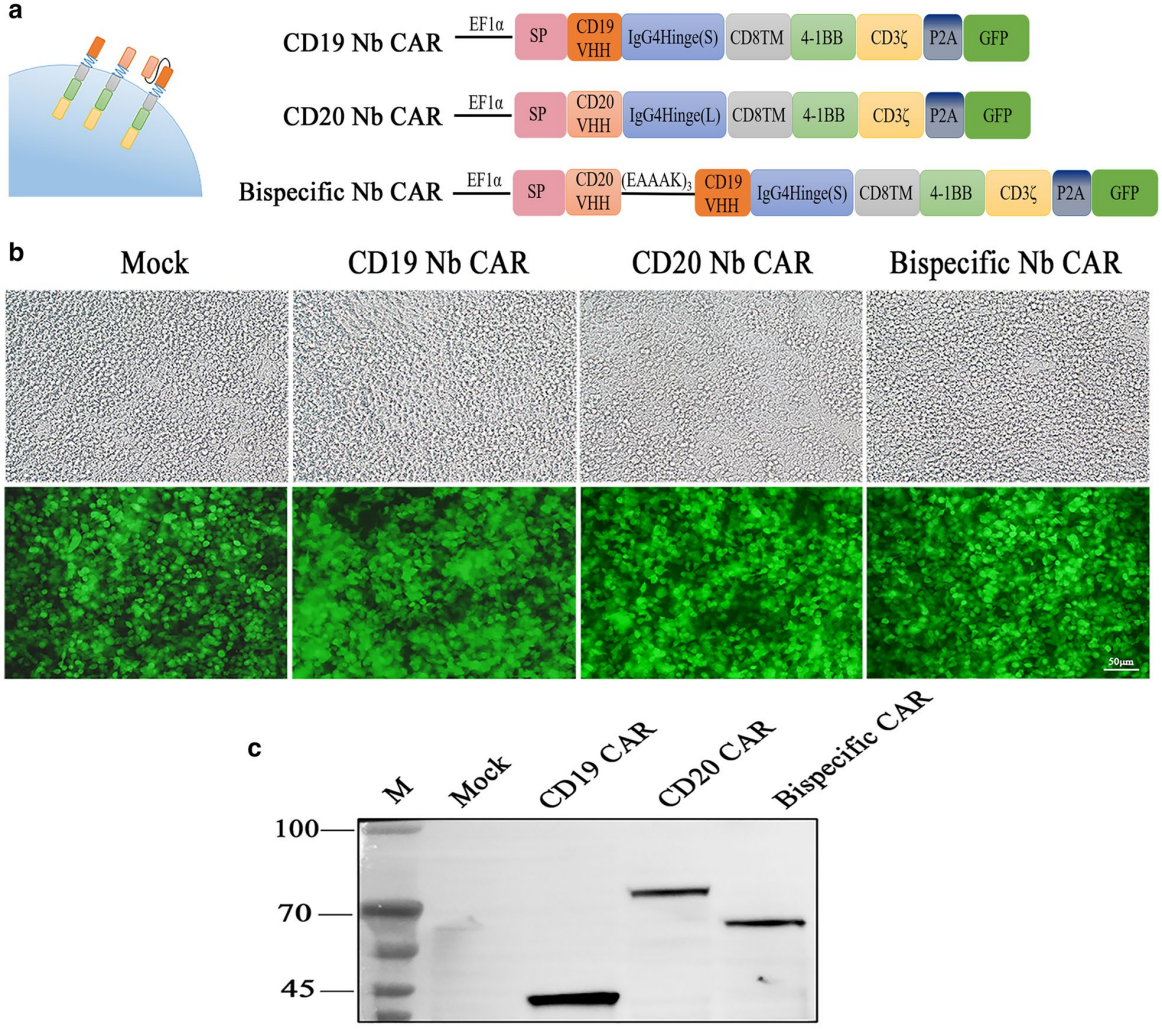
Nb CAR construction.  **A **Schematic diagram of Nb CARs, including the CD8 signal peptide (SP), Nb, IgG4 hinge, CD8TM, 4-1BB, and CD3ζdomain. **B**, **C** Nb CARs expression on 293T cells. The 293T cells were transduced with control or different Nb CARs, GFP expression was observed after 72 h, cell lysates were collected and blotted with the primary antibody mouse anti-human CD3, and specific bands were observed

Similar to conventional CAR constructs, all CARs we generated here were composed of three parts: binding domains, hinge and transmembrane domains, intracellular signalling domains (Fig. 2A). The lentiviral vector encoding only GFP was used as a control. To assess whether the Nb CARs were successfully constructed, we delivered lentivirus to 293T cells and observed high levels of GFP expression (Fig. 2B). Then, the cell proteins were collected to detect the molecular weights of the CAR proteins by probing of CD3ζ. Western blot analysis showed specific bands at approximately 40 kDa, 70 kDa, and 60 kDa, the expected target band sizes of the CD19 Nb CAR, CD20 Nb CAR, and Bispecific Nb CAR, respectively, which proved that the CAR gene fragment had been integrated into 293 T cells (Fig. 2C). Thus, we successfully constructed three nanobody-based CARs.

**Fig. 3:**
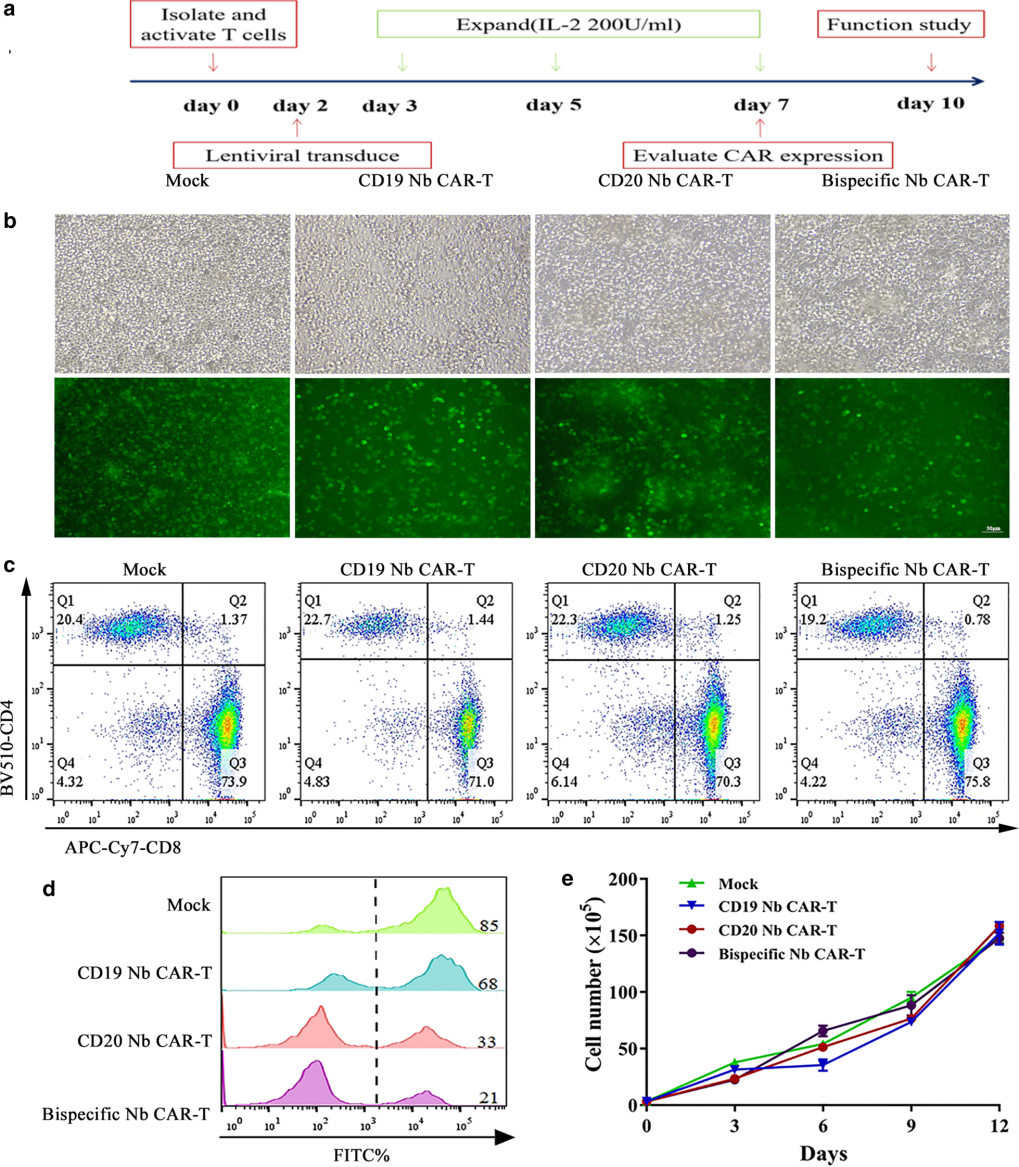
Nb CAR-T cell generation and expansion. **A **Schematic diagram of Nb CAR-T cells generation. Primary T cells were activated, transduced and then expanded with IL-2 up to day 10. **B** The bright field and fluorescence field in different groups were observed by fluorescence microscopy. **C**, **D** Nb CAR-T expression and phenotype. Modified T cells were collected on day 7, T cells were stained with 7-AAD, PE-conjugated mouse anti-human CD3, APC-Cy7^TM^-conjugated mouse anti-human CD8, and BV510-conjugated mouse anti-human CD4 (BD), and CAR-T cells positive expression was defined as FITC + PE+. The dead cells were excluded by 7AAD. **E** The expansion of Nb CAR-T cells. The cell numbers were recorded every 3 days after transduction on day 3

Then CAR-T cells were generated and expanded as described in Fig. 3A. Before transduction, we optimized the culture and expansion system of primary T cells. We found that T cells stimulated with immobilized CD3/CD28 for 48 h were supplanted with 10% autologous plasma, and 200 U/ml IL-2 resulted in the best proliferation (see Additional file 1). At 72 h post transduction, we observed that all groups had bright GFP signals (Fig. 3B). The CD8 to CD4 ratio remained equal among the groups (Fig. 3C), and the positive rate of Nb CAR-T cells ranged from 20 to 80% (Fig. 3D). Additionally, the numbers of Nb CAR-T cells were recorded, and the Nb CAR-T cells showed a similar expansion ability compared to that of the mock group, with a nearly 150-fold increase after transduction (Fig. 3E). Thus, we successfully prepared Nb CAR-T cells for a subsequent assay study.

### Nb CAR-T cells specifically recognize target cells in vitro

**Fig. 4:**
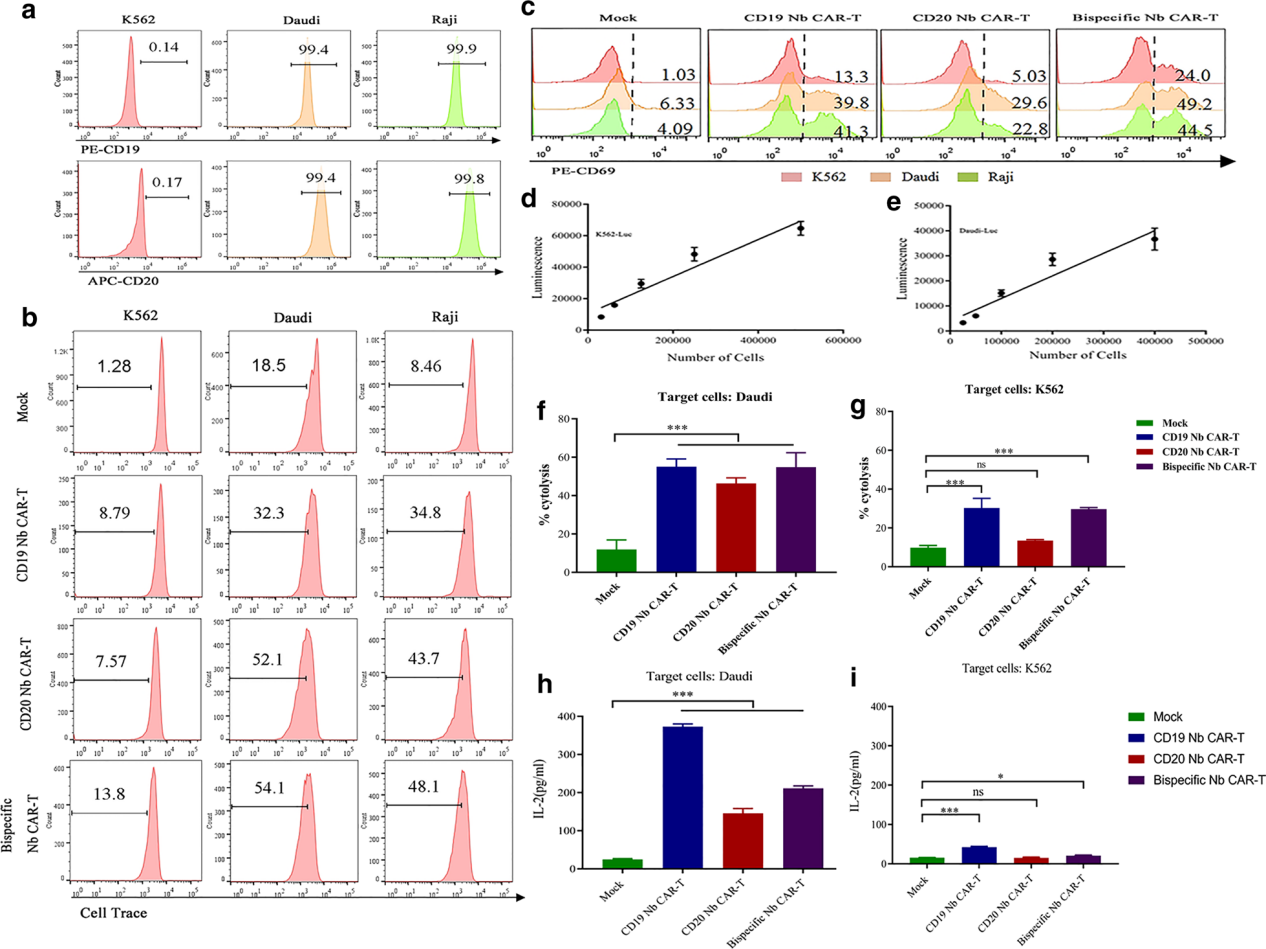
Nb CAR-T cells specifically proliferate, activate and kill target cells.  **A **The expression of CD19 and CD20 on Raji, Daudi, and K562 cells. **B** The proliferation of Nb CAR-T cells. Cell Trace-labelled Nb CAR-T cells were incubated with mitomycin C-treated Daudi, Raji or K562 cells for 5 d, and then, the Cell Trace dilution was analysed by flow cytometry. CAR-T cells were gated on GFP signals. **C** The expression of CD69 on Nb CAR-T cells. Nb CAR-T cells were incubated with Daudi, Raji and K562 cells for 24 h, and then, the T cell activation marker CD69 was detected by flow cytometry. **D**, **E** The generation of Daudi-luc and K562-luc cell lines. Daudi and K562 cells were transduced with lentivirus encoding the luciferase gene, followed by puromycin selection. **F**, **G** Cytotoxicity of Nb CAR-T cells. Nb CAR-T cells were incubated with Daudi, K562 cells at an E:T of 2:1, and cell viability was determined via bioluminescence. **H**, **I** Cytokine production of Nb CAR-T cells. Nb CAR-T cells were incubated with Daudi or K562 cells overnight, and then, the culture supernatant was collected to measure IL-2. Data were analysed by one-way ANOVA. *p < 0.05, **p < 0.01, ***p < 0.001, ns, not significant (p > 0.05)

To evaluate whether these Nb CAR T cells can specifically recognize target tumour cells, we first used tumour cell lines as targets. Raji and Daudi cells were used as positive target cells because they endogenously express both CD19 and CD20, and K562 cells were used as a negative control because they lack these antigens (Fig. 4A).

Using Cell Trace Far Red, we evaluated the proliferative potential of the Nb CAR-T cells in response to tumour cells. The results revealed that the Nb CAR-T cells underwent robust proliferation in response to Raji and Daudi cells but not K562 cells.

In contrast, the mock group had a lower proliferative potency than that of the Nb CAR-T cells (Fig. 4B). In addition, the cells were collected for CD69 staining after 24 h of coincubation, similar to the protocol for proliferation. Flow cytometry showed that both Raji and Daudi cells, but not K562 cells, could stimulate Nb CAR-T cell activation, with CD69 expression upregulated in all Nb CAR-T cell groups (Fig. 4C). Thus, the Nb CAR-T cells can react with target cells in vitro in an antigen-dependent way.

### Nb CAR-T cells specifically kill target cells in vitro

To determine whether Nb CAR-T cells possess the functional properties that specifically kill target cells in vitro, we assessed cytotoxicity and cytokine release. We first engineered cell lines that stably expressed luciferase by transferring the firefly luciferase gene to K562 and Daudi cells (Fig. 4D, E). Similar to the results of the activation and proliferation assays, Nb CAR-T cells killed Daudi-luc cells but were incapable of killing K562-luc cells, and mock T cells had only slight cytotoxicity to tumour cells (Fig. 4F, G). ELISA results showed that the levels of IL-2 in the supernatants of the cocultured Nb CAR-T cells and Daudi-luc cells were obviously higher than those of the mock group (Fig. 4H, I). Thus, we concluded that the Nb CAR-T cells have robust activity against tumour cells in vitro*.*

### Nb CAR-T cells can recognize and kill primary ALL tumour cells in vitro

**Fig. 5:**
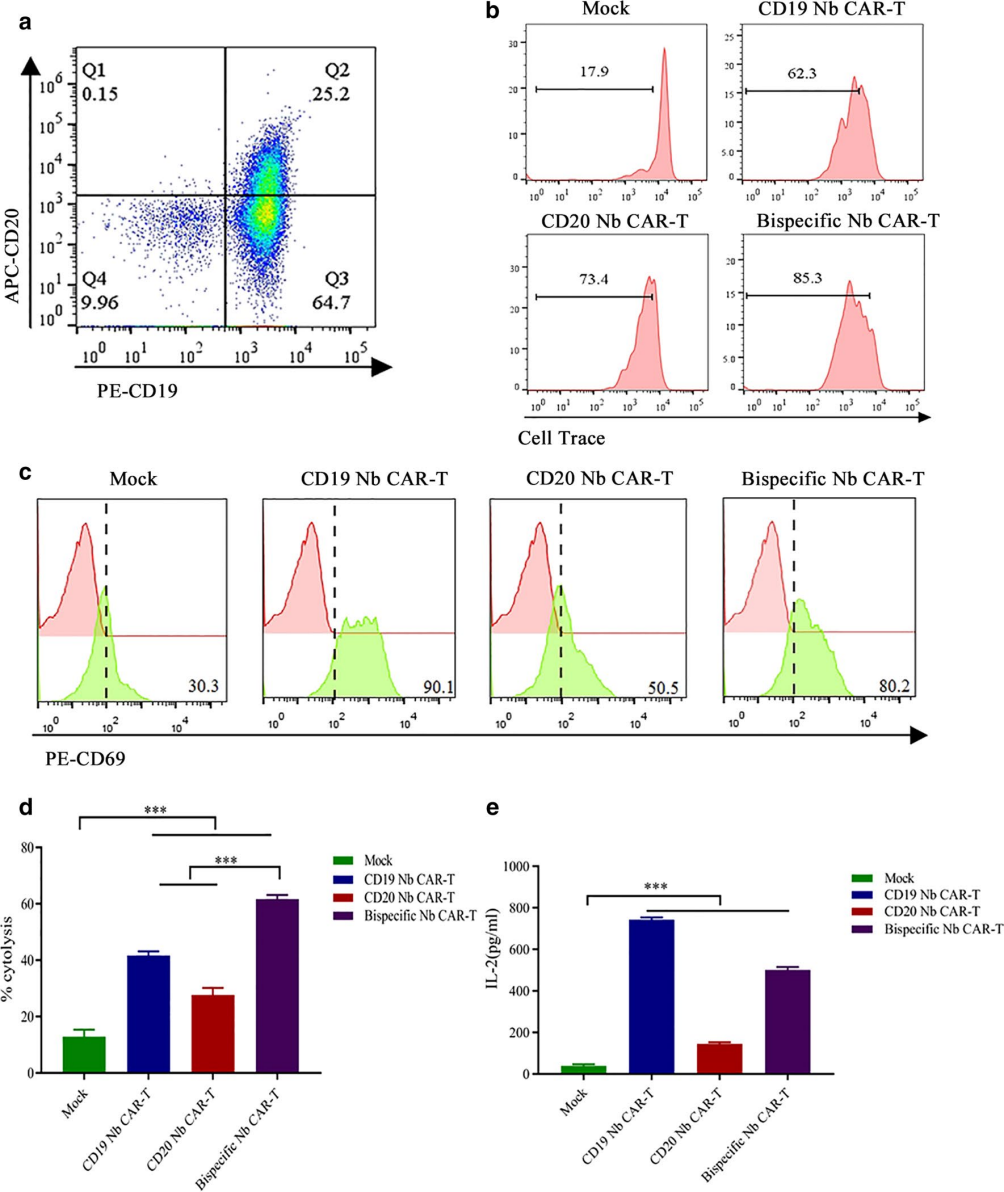
Nb CAR-T cell cytotoxicity on patient-derived tumour cells. **A** Expression of CD19 and CD20 in patient samples. Patient PBMCs were harvested and stained with PE-conjugated mouse anti-human CD19 (BD) and APC-conjugated mouse anti-human CD20 (BD) at room temperature for 30 min, washed and resuspended in FACS buffer, Flow cytometry was used to assess CD19 and CD20 expression. **B**, **C** The proliferation and activation of Nb CAR-T cells stimulated by primary ALL tumour cells. Nb CAR-T cells and tumour cells were coincubated overnight or 5 days, followed by Flow cytometry analysis. **D** Nb CAR-T cell cytotoxicity to primary ALL tumour cells. Nb CAR-T cells were incubated with primary tumour cells, and LDH-based cytotoxicity assays were performed. **E** Cytokine production of Nb CAR-T cells to primary tumour cells. Nb CAR-T cells and primary tumour cells were coincubated overnight, and then, the expression of IL-2 was determined by ELISAs. Data were analysed by one-way ANOVA. *p < 0.05, **p < 0.01, ***p < 0.001, ns, not significant (p > 0.05)

Based on the above results, we wondered whether these Nb CAR-T cells also have potential cytotoxicity to primary tumour cells. We collected primary tumour cells from PD-ALL samples, and the flow cytometric results showed that a majority of the lymphocyte population expressed CD19 and that approximately 20% expressed CD20 (Fig. 5A); additional information is described in (see Additional file 2: Table S1). Consistent with the cytotoxicity assay using cell lines as targets, after 5 days of incubation with Nb CAR-T cells, we found that PD-ALL cells could stimulate Nb CAR-T cells proliferation (Fig. 5B). We also observed increased expression of the activation marker CD69 (Fig. 5C). Furthermore, we conducted an LDH assay to estimate cytotoxicity, and the results showed that PD-ALL cells were lysed when incubated with Nb CAR T cells rather than mock T cells (Fig. 5D), and elevated IL-2 was also observed (Fig. 5E). Together, these results demonstrated that Nb CAR-T cells could specifically recognize and kill primary ALL tumor cells in vitro*.*

## Discussion

CAR-T cell immunotherapy has achieved remarkable success in haematologic malignancy, but more efforts to expand CAR-T-cell applications are needed [44–50]. Studies have commonly attempted to improve efficacy, overcome off-tumour toxicities and optimize CAR designs [51–55]. Currently, nanobodies show a promising future in CAR-T therapy due to their inherent advantages compared to mAbs [56–59]. First, researchers could enhance the specificity of CAR-T cells by developing CAR constructs that simultaneously target multiple antigens and elicit appropriate immunological synapses due to their monomeric format, small size and moderate aggregation. Then, researchers could improve the efficiency of CAR-T cells by simultaneously isolating multiple nanobodies that preferentially bind various epitopes of the same antigen. In addition, CAR-T cell cytotoxicity against solid tumours could be enhanced due to its strong penetration into tissue, and related targets include CD105, PD-L1 and EIIIB splice variants of fibronectin [23, 60].

Here, using our prior natural Nb-expressing phage display library (a low cost, broadly availability approach compared to immune and synthetic libraries), we selected human CD19- and CD20-specific nanobodies. Preliminary analysis showed that these nanobodies could bind the CD19 and CD20 antigens with high affinity. These encouraging results prompted us to determine whether CAR-T cells prepared with nanobodies could effectively kill tumour cells and whether bispecific Nb CAR-T cells could also be prepared simultaneously. During the design of the CAR structure, we used 4-1BB as a costimulatory domain rather than CD28, which tended to persist for a shorter time due to the low TSCM and high exhaustion phenotype [61–63]. Given that CD20 is classified as a transmembrane protein, we selected a long hinge in the CD20 CAR. For bispecific Nb CAR-T cells, we used (EAAAK)3 rather than conventional (GGGGS)3 as the linker, which can easily mispair and aggregate. Furthermore, considering the structural and positional characteristics of the CD19 and CD20 proteins on tumour cells, we linked CD20 at the N-terminal side of the CAR and CD19 close to the cell membrane [43].

Then, before exploring the function of our prepared Nb CAR-T cells, we optimized the T cell culture conditions. T cells that stimulated both RetroNectin and anti-human CD3/CD28 antibodies had obvious proliferation in the first few days, followed by a decline, while T cells continued to expand after stimulation with anti-human CD3/CD28 antibodies alone. We concluded that overactivity may lead to cell death. Therefore, we only used anti-human CD3/CD28 antibodies as stimuli.

When Nb CAR-T cells were coincubated with tumour cells, we found that the Nb CAR-T cells were specifically activated by tumour cells, showing robust proliferation, cytokine release and antitumour efficiency, which were consistent with recent studies. In addition, Xin He reported that bispecific and split CAR-T cells that target TIM-3 and CD13 (highly expressed in AML) could eliminate AML [22]. Recent studies also showed that knockout of PD-1 by CRISPR/Cas9 improved the efficiency of CAR-T cells [64]. Therefore, we reasoned that combining Nb CAR-T cells with editing technology or pharmacological intervention could be a promising strategy against tumour cells.

Furthermore, to determine the functions of these Nb CAR-T cells against primary tumour cells, we obtained samples from ALL patients. Notably, the results showed that these Nb CAR-T cells could also recognize primary tumour cells and potently eliminate tumour cells. All of these results confirmed that Nb CAR-T cells have potential cytotoxicity to tumour cells. Although nanobodies have low immunogenicity, some unfavourable results have been reported. Justyna Jureczek et al. found that murine and humanized scFv-based CAR-T cells possessed significantly superior efficiency compared to Nb-based CAR-T cells [65]. Therefore, we plan to construct humanized Nb CAR-T cells and evaluate their functions in future work.

Although we acquired promising results, several limitations should be noted. First, more nanobodies that target CD19 and CD20 should be panned to select the best sequence for CAR design. Furthermore, CAR-T cell biological properties, such as memory phenotype and degree of exhaustion, should be improved. Third, more patient samples need to be used. Finally, further study needs to be carried out in vivo to verify the substantial antitumour effects of Nb CAR-T cells.

## Conclusions

In conclusion, we successfully generated Nb CAR-T cells and demonstrated their robust activity against tumour cell lines and patient primary tumour cells. Our study indicates that a natural Nb-expressing phage display library can also generate binders with efficacy against related targets, demonstrates that nanobody-engineered T cells have potential cytotoxicity to tumour cells, and highlights the promising broad utility of nanobodies in CAR-T cells and perhaps CAR-NK and CAR-γδT cells.

## Supplementary Information

**Additional file 1: Figure S1.** Optimization culture condition of T cells. (A-B) Cell morphology under different conditions. Both EDTA and heparin sodium anticoagulant whole blood were used, T cells were purified by positive selection and divided into different treatments. (A) T cells were stimulated by anti-human CD3, CD28 antibodies and cultured in different systems for 24h. (B) T cells were stimulated by anti-human CD3, CD28 antibodies alone or with Retronectin for 48h, 72h, then cultured in different conditions. (C) Cells expansion (heparin sodium anticoagulant whole blood). The numbers of T cells in some groups were recorded on day 7.

**Additional file 2: Table S1.** Clinical information of patient samples. Patient characteristics and other clinical information of primary ALL patient samples.

## Data Availability

The datasets used and analysed in the current study are available from the corresponding author in response to reasonable requests.

## Abbreviations

CAR-T: Chimeric antigen receptor T cells
Nb: Nanobody
Nb CAR-T: Nb-based Chimeric antigen receptor T cells
B-ALL: B-cell precursor acute lymphoblastic leukaemia
PD-ALL: Patient-derived tumour cells of acute lymphoblastic leukaemia
AML: Acute myeloid leukaemia
mAb: Monoclonal antibody
scFv: Single chain variable fragment
VHH: Variable domain of heavy chain of heavy chain antibody
HcAbs: Heavy chain antibodies
CDR: Complementarity-determining region
QDs: Fluorescent semiconductor quantum dots
SPR: Surface plasmon resonance
PD-1: Programmed cell death protein 1
TIM-3: T cell immunoglobulin-3
