# Supplementary figures and images for: Nanobody-armed T cells endow CAR-T cells with cytotoxicity against lymphoma cells
Source: Cancer Cell Int. 2021 Aug 24;21:450. doi: 10.1186/s12935-021-02151-z (PMC8386010; doi:10.1186/s12935-021-02151-z)

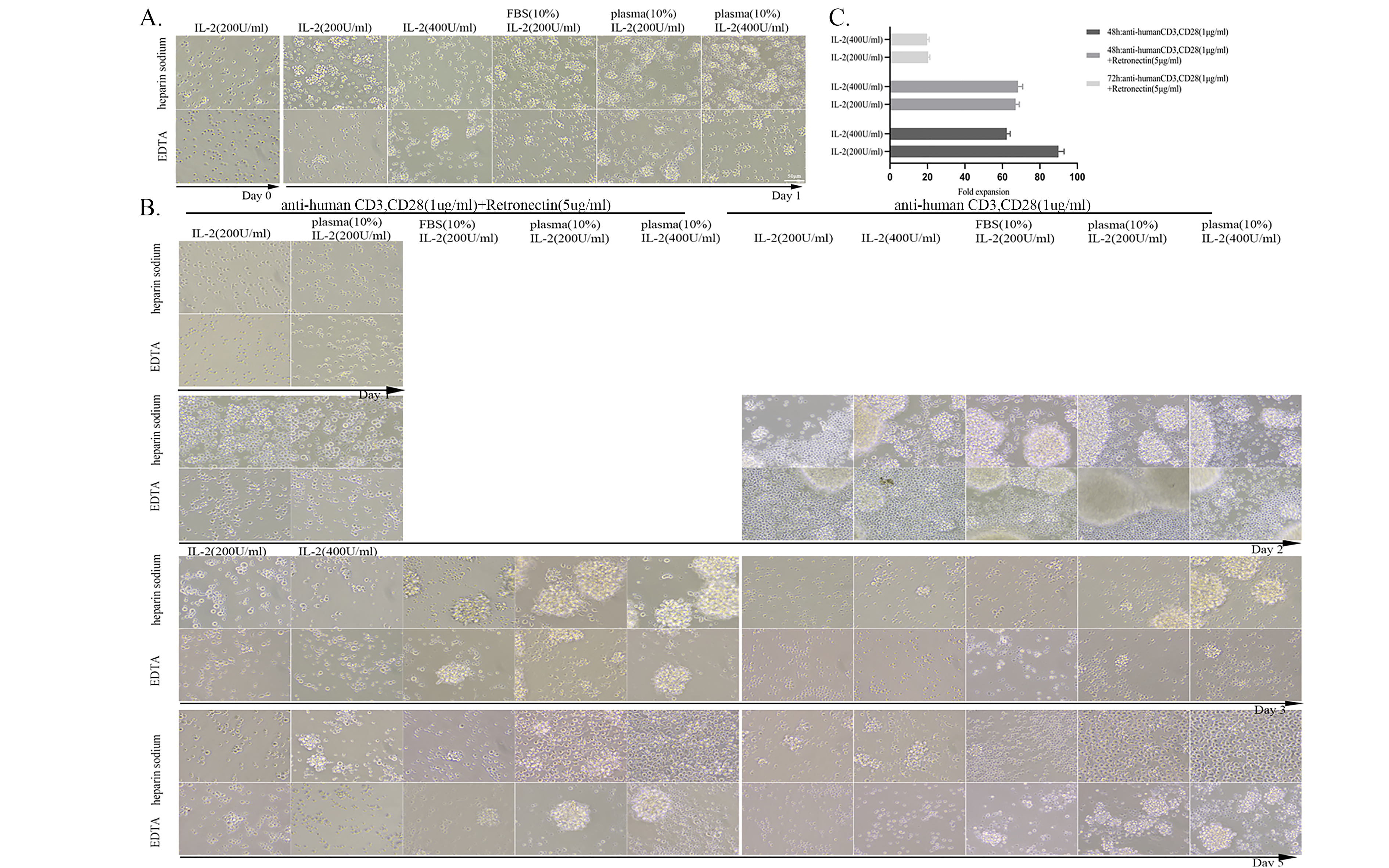

Supplement: Supplementary file 1 — Additional file 1: Figure S1. Optimization culture condition of T cells. (A-B) Cell morphology under different conditions. Both EDTA and heparin sodium anticoagulant whole blood were used, T cells were purified by positive selection and divided into different treatments. (A) T cells were stimulated by anti-human CD3, CD28 antibodies and cultured in different systems for 24h. (B) T cells were stimulated by anti-human CD3, CD28 antibodies alone or with Retronectin for 48h, 72h, then cultured in different conditions. (C) Cells expansion (heparin sodium anticoagulant whole blood). The numbers of T cells in some groups were recorded on day 7. [file 12935_2021_2151_MOESM1_ESM.tif]
