# Supplementary material for: Nanobody-armed T cells endow CAR-T cells with cytotoxicity against lymphoma cells
Source: Cancer Cell Int. 2021 Aug 24;21:450. doi: 10.1186/s12935-021-02151-z (PMC8386010; doi:10.1186/s12935-021-02151-z)
Supplement: Supplementary file 2 — Additional file 2: Table S1. Clinical information of patient samples. Patient characteristics and other clinical information of primary ALL patient samples. [file 12935_2021_2151_MOESM2_ESM.docx]

Clinical information of patient sample

| Sex | Age | Disease | Sample | Immunophenotype | Karyotype analysis | Fish | Fusion gene |
| --- | --- | --- | --- | --- | --- | --- | --- |
| Female | 10 | B-ALL | PBMC | Lymphocyte 4%,  Myeloid 0.9%,  Abnormal cell 95.1%  (HLA-DR+CD10+  CD19+CD22+  CD34+TdT+/partial expression of CD20+CD38+ cIgM) | 46, XX,del (9)(p13),t(9;22)(q34;q11)[13]/46,idem,del(3)(q21),add(5)(q33),add(7)(p15) [7] | nuc ish (ABL1×2) (5'ABL1 sep 3'ABL1×1) [316/400] | BCR-ABL1 |

Patient characteristics and other clinical information of primary ALL patient sample.
